# Association of potentially inappropriate medications with prognosis among older patients with non-small cell lung cancer

**DOI:** 10.1186/s12877-024-05138-3

**Published:** 2024-06-25

**Authors:** Zhuo Ma, Man Xu, Mengyuan Fu, Tao Huang, Luwen Shi, Yuhui Zhang, Xiaodong Guan

**Affiliations:** 1 Department of Pharmacy Administration and Clinical Pharmacy, School of Pharmaceutical Sciences, Peking University, Beijing, 100191 China; 2 Department of Pharmacy, Beijing Chao-Yang Hospital, Capital Medical University, Beijing, 100020 China; 3 Department of Pharmacy, Beijing Obstetrics and Gynecology Hospital, Capital Medical University, Beijing, 100010 China; 4 International Research Center for Medicinal Administration, Peking University, #38 Xueyuan Road, Haidian District, Beijing, 100191 China; 5 Department of Respiratory and Critical Care Medicine, Beijing Chao-Yang Hospital, Beijing Institute of Respiratory Medicine, Capital Medical University, No. 8 Gongtinan Road, Chaoyang District, Beijing, 100020 China

**Keywords:** Non-small cell lung cancer, Potentially inappropriate medications, Older, Overall survival

## Abstract

**Background:**

Potentially inappropriate medications (PIMs) are common among older adults with cancer, but their association with overall survival (OS) among non-small cell lung cancer (NSCLC) patients remains unclear. This study aimed to investigate the association between the use of PIMs and OS in patients with NSCLC.

**Methods:**

In this cohort study, we included patients ≥ 65 years with newly diagnosed NSCLC from January 2014 to December 2020. Potentially inappropriate medication (PIM) is defined by the Beers criteria of 2019 at baseline and within six months following the initiation of systemic therapy. Multivariable Cox regression model was built to assess the association between PIMs and overall survival (OS).

**Results:**

We finally included 338 patients with a median follow-up for OS of 1777 days. The prevalence of patients receiving at least one PIM was 39.9% (135/338) and 61.2% (71/116) at baseline and after systemic therapy, respectively. The most important factor associated with PIM use was the number of prescribed medications (*P* < 0.001). Baseline PIM use and PIM after systemic therapy were significantly associated with inferior OS (476 days vs. 844 days, *P* = 0.044; and 633 days vs. 1600 days, *P* = 0.007; respectively). In multivariable analysis, both baseline PIM use and PIM after systemic therapy were independent predictors of poor prognosis (adjusted HR, 1.33; 95% CI, 1.01–1.75; *P* = 0.041; and adjusted HR, 1.86; 95% CI, 1.11–3.14; *P* = 0.020; respectively).

**Conclusions:**

PIMs are prevalent among older patients with NSCLC and are independent predictors of NSCLC prognosis. There is an urgent need for clinicians to conduct medication reconciliation and appropriate deprescribing for this population, especially for patients with multiple PIMs.

**Supplementary Information:**

The online version contains supplementary material available at 10.1186/s12877-024-05138-3.

## Background

Lung cancer is a leading cause of cancer-related deaths across the world [1] and non-small cell lung cancer (NSCLC) is the most frequent histological subtype of lung cancer (approximately 80–85%) [2]. Currently, the median age of patients with lung cancer at their diagnosis is around 70 and almost 10% of patients are ≥ 80 years old [3]. With populations aging, the incidence of lung cancer among the elderly is expected to further increase in near future [4].

Potentially inappropriate prescribing, which has become a global concern particularly among the elderly, refers either to: (1) potentially inappropriate medications (PIMs), the use of drugs where no clear clinical indication exists (overprescribing) or the use of an indicated drug where the risk outweighs the benefit or when a safer or more effective alternative is available (misprescribing) or (2) potential prescribing omissions (PPOs), i.e., not prescribing a beneficial medicine for which there is a clear clinical indication (underprescribing) [5–7].

PIM use is a common problem in the general geriatric population, but it may imply more critical considerations for the care of elderly cancer adults who tend to have developed many chronic health conditions, in addition to cancer, and require multiple therapeutic regimens to be prescribed simultaneously [8]. Elderly cancer patients also require additional medications to manage treatment-related symptoms or adverse reactions [9, 10]. Furthermore, as cancer patients are often treated by multiple physicians, they may receive medications for overlapping indications [11]. The National Comprehensive Cancer Network Guidelines for Senior Adult Oncology [12] recommend the use of evidence-based instrument to determine the appropriateness of medication prescribed, such as the Beers Criteria [13], the Screening Tool of Older Persons’ Prescriptions (STOPP), the Screening Tool to Alert to Right Treatment (START) criteria [7], and the medication appropriateness index (MAI) [14].

PIM use is also concerning as it may be associated with the prognosis of cancer patients. However, evidence on the safety of PIM use among elderly cancer patients is still sparse and conflicting [15, 16]. A latest systematic review has found that the pooled effect estimate of six cohort studies indicated a 43% increased mortality among PIM users [16].

Although a previous study has reported an incidence of PIM as high as 35% in older patients with lung cancer [17], the impact of PIM use on the clinical outcomes, such as survival, of lung cancer patients have not yet been well investigated, which hinders the understanding of its significance. Since the overall prevalence of PIM use among older patients with lung cancer increased overtime in China [18] and PIM use may be associated with negative clinical outcomes, it is of special importance to extensively investigate the association between PIM use and overall survival (OS) in lung cancer patients. Therefore, in this cohort study, we sought to investigate the prevalence and related risk factors of PIM use in older NSCLC patients and evaluate whether PIM use at baseline or after systematic therapy in NSCLC patients is an independent predictor of OS.

## Methods

### Study population

Patients with newly diagnosed NSCLC among an observational cohort at Beijing Chao-Yang Hospital from January 2014 to December 2020 were enrolled. The inclusion criteria were as follows: (1) diagnosis of NSCLC by cytological or histological examination; and (2) aged 65 and older. Patients without any medications at the time of diagnosis and loss to follow-up were excluded. Permission for data analysis was approved by the Ethics Committee of the Beijing Chao-Yang Hospital, Beijing Institute of Respiratory Medicine, Capital Medical University, Beijing, China (No. 2009–4, 2016–79 and No. 2021-ke-443), and written informed consent was obtained from all patients.

### Assessment of PIMs

Prescribed drugs were retrospectively extracted from patients’ electronic medical records (EMRs) and were analyzed at two time points, at the time of patients’ diagnosis of NSCLC and during the first six months after the initiation of their cancer therapy.

Whether medications shall be categorized into PIMs were assessed based on the 2019 Beers criteria [13]. Two authors (Zhuo Ma and Man Xu) independently reviewed each patient’s electronic records and identified the use of PIMs. Each reviewer was blinded to the other reviewer in the process of data extraction and identification of PIMs. Another author, Yuhui Zhang, was consulted if there were any discrepancies. For compound drugs, each active component was counted separately. Topical medications were not counted in the number of medications.

### Assessment of clinical outcome

The clinical outcome was OS, which was collected from patients themselves via telephone follow-up. OS was defined as the time between the day of first diagnosis of NSCLC to the day of death, loss to follow-up, or censoring (November 30, 2022), whichever was earliest.

### Covariates

Independent variables were extracted from EMRs as follows: sex; age; body mass index (BMI); smoking status; Eastern Cooperative Oncology Group–Performance Status (ECOG-PS); tumor stage (TNM of the International Association for the Study of Lung Cancer (version 8) [19]; the number of medications; and surgery. The Charlson Comorbidity Index (CCI) [20] score was calculated for each patient to measure the burden of comorbidities.

### Statistical analysis

Descriptive analyses were conducted to describe patient characteristics. The Student’s t-test or non-parametric test was applied to compare continuous variables for the mean or median, respectively. The chi-squared test was used for between-group comparisons of categorical variables. Logistic regression model was constructed to evaluate the association between the use of PIMs and covariates. Variables including age, sex, BMI, PS, CCI, and the number of medications prescribed were used in the analysis of predictors of PIM use [21]. Differences in survival between groups were derived from the Kaplan-Meier (KM) analysis and Log-rank testing. Multivariable Cox proportional hazards (PH) regression model was performed to determine whether baseline PIM use and PIM use after systemic therapy were independent predictors affecting survival. Model 1 was adjusted for variables that were associated with PIM use at the significance level of 0.1 in the univariate analysis. Model 2 was adjusted for variables based on both statistical and clinical significance. Results were expressed in hazard ratios (HRs) and 95% confidence intervals (CIs). The proportional hazards assumption was assessed by the Schoenfeld residuals test. P-values less than 0.05 were considered to be statistically significant and all tests were two-sided. The statistical analysis was carried out using SPSS software (version 23.0).

## Results

### Patient characteristics

Table 1 illustrates the characteristics of the study participants. In total, 338 patients were included in the study, consisting of 131 females (38.8%) and 207 males (61.2%). The median age of the cohort was 70 years (IQR, 67–74) and the mean BMI was 24 kg/m^2^ (SD, 3.4). Only 26.9% of the patients exhibited poor ECOG PS and almost half of the patients (46.7%) never smoked. In terms of tumor stage, 73.1% of the patients were classified as stage IIIB-IV. The median (IQR) CCI points and prescribed medications at baseline were 2 (1–6) and 7 (4–11), respectively.

**Table 1:** Patient characteristics at baseline and within six months following the initiation of systemic therapy

Patient characteristics	Baseline	Within six months following the initiation of systemic therapy	*P* value^a^
**Overall**	338	116	
**Age (median and IQR)**	70(67–74)	70(67–73)	0.328
65–74	256 (75.7%)	92(79.3%)	
≥ 75	82 (24.3%)	24(20.7%)	
**Sex**			0.738
Male	207(61.2%)	69(59.5%)	
Female	131(38.8%)	47(40.5%)	
**BMI, kg/m** ^**2**^ **(mean ± SD)**	24.0 ± 3.4	24.0 ± 3.9	0.949
**Smoking history**			0.776
Yes	180(53.3%)	60(51.7%)	
No	158(46.7%)	56(48.3%)	
**ECOG PS**			0.252
0–2	247(73.1%)	91(78.4%)	
3–4	91(26.9%)	25(21.6%)	
**CCI** **(median and IQR)**	2(1–6)	3(1–7)	0.530
**Tumor stage**			0.087
I-IIIA	91(26.9%)	22(19.0%)	
IIIB-IV	247(73.1%)	94(81.0%)	
**Number of Medications** **(median and IQR)**	7(4–11)	13(10–16)	< 0.001
1–4	93(27.5%)	7(6.0%)	
≥ 5	245(72.5%)	109(94.0%)	
**Surgery**			0.128
Yes	91(26.9%)	23(19.8%)	
No	247(73.1%)	93(80.2%)	

*Note* SD, standard deviation; IQR, interquartile range; BMI, body mass index; ECOG PS, Eastern Cooperative Oncology Group Performance Status; CCI, Charlson Comorbidity Index

^a^ P value tests for differences in patients’ characteristics between at baseline and within six months following the initiation of systemic therapy

The number of drugs taken by patients after the initiation of systemic treatment increased significantly to 13(10–16) (*p* < 0.001).

### PIM use at baseline

At baseline, a total of 209 PIMs were identified. PIMs were detected for 39.9% (135/338) of the patients, among whom 90 (66.7%) used one PIM and 45 (33.3%) used two or more PIMs. (Supplementary Table 1)

Among the top five PIMs identified based on the Beers criteria, diuretics (*N* = 57, 27.3%) accounted for the most frequently used PIM at baseline, followed by benzodiazepines (*N* = 29, 13.9%), tramadol (*N* = 20, 9.6%), insulin, sliding scale (*N* = 19, 9.1%), non–cyclooxygenase-selective non-steroidal anti-inflammatory drugs (NSAIDs) (*N* = 10, 4.8%), and proton-pump inhibitors (PPIs) (*N* = 10, 4.8%; see Supplementary Table 2 for details).

### PIM use within six months following the initiation of systemic therapy

After the initiation of systemic therapy, a total of 116 patients with 124 PIM use were identified. Compared with baseline, the frequency of PIM use during the time interval of 0–6 months after the initiation of systemic therapy was higher, with 61.2% of the patients (71/116) using at least one PIM (*P* < 0.001). Among patients who used PIMs, 34 (47.9%) used one PIM and 37 (52.1%) had two or more PIMs (see Supplementary Table 1 for details).

Consistent with PIM use at baseline, diuretics (*N* = 37, 29.8%), benzodiazepines (*N* = 13, 10.5%), and non–cyclooxygenase-selective NSAIDs (*N* = 7, 5.6%) were among the top five frequently used PIMs. In contrast, first-generation antihistamines (*N* = 23, 18.5%) and metoclopramide (*N* = 15, 12.1%) were frequently used PIMs only after the initiation of systemic therapy (Supplementary Table 2).

### Factors associated with PIMs

Multivariate analysis showed that PIM use at baseline and after the initiation of systemic therapy were both associated with the number of prescribed medications (OR: 1.20 [95% CI: 1.14–1.27], *p* < 0.001; and OR: 1.24 [95% CI: 1.12–1.37], *p* < 0.001; respectively) (Supplementary Table 3).

### Association between PIMs at baseline and overall survival

**Fig. 1:**
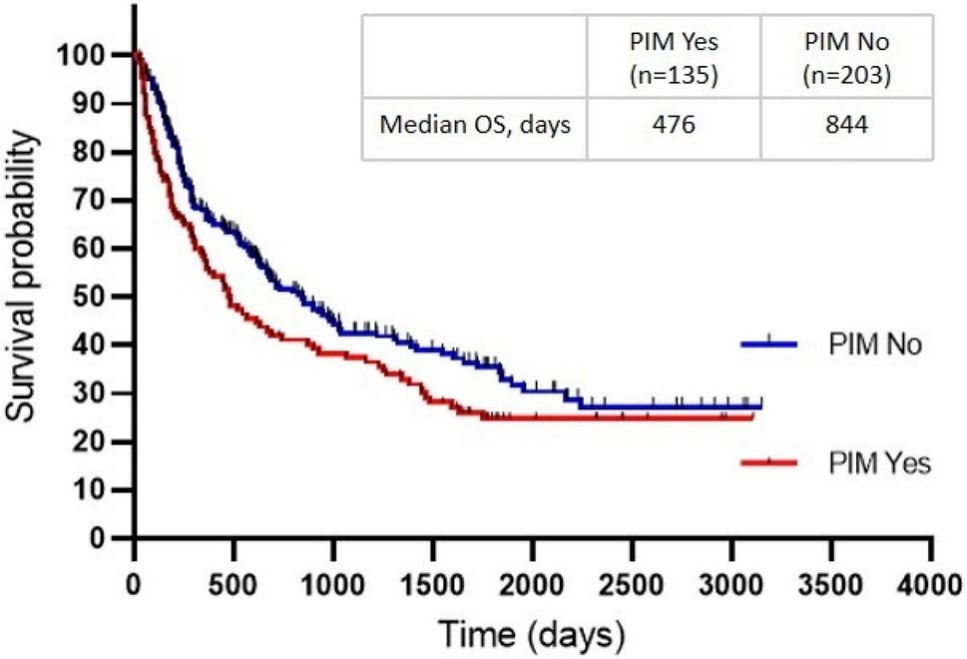
The Kaplan-Meier analysis of overall survival in NSCLC patients with or without PIM use at baseline (*P* = 0.044). *Note* PIM, potentially inappropriate medication; OS, overall survival; NSCLC, non-small cell lung cancer

A total of 338 patients were available for outcome analysis. The median follow-up for OS was 1,777 days (1,734-1,820 days), during which 213 patients deceased (63%). The Kaplan–Meier survival analyses showed that patients who used at least one PIM at baseline had significantly worse survival compared with those who did not use PIMs (median, 476 days vs. 844 days, *P* = 0.044) (Fig. 1).

In multivariate analysis model 1, baseline PIM use was an independent risk factor for compromised OS (adjusted HR, 1.33; 95% CI, 1.01–1.75; *P* = 0.041). Results were similar in model 2 when controlled for age, sex, BMI, smoking history, tumor stage, ECOG PS, CCI, and the number of medications (adjusted HR, 1.35; 95% CI, 1.01–1.80; *P* = 0.046). The use of two or more PIMs remained an independent predictor of poorer OS (multivariate analysis model 1: adjusted HR, 1.79; 95% CI, 1.23–2.63; *P* = 0.003; multivariate analysis model 2: adjusted HR, 1.78; 95% CI, 1.20–2.65; *P* = 0.005), whereas the use of one PIM only was not (multivariate analysis model 1: adjusted HR, 1.15; 95% CI, 0.83–1.58; *P* = 0.400; multivariate analysis model 2: adjusted HR, 1.17; 95% CI, 0.84–1.64; *P* = 0.361). (Table 2)

**Table 2:** Univariate and multivariate Cox regression analyses of association between baseline PIM use with overall survival

Variables	Univariate analysis		Multivariate model 1^a^		Multivariate model 2^b^	
	HR (95% CI)	*P* value	HR (95% CI)	*P* value	HR (95% CI)	*P* value
PIM (yes vs. no)	1.32 (1.01–1.73)	0.045	1.33 (1.01–1.75)	0.041	1.35 (1.01–1.80)	0.046
Number of PIMs (1 vs. 0)	1.80 (0.79–1.49)	0.626	1.15 (0.83–1.58)	0.400	1.17 (0.84–1.64)	0.361
Number of PIMs (≥ 2 vs. 0)	2.04 (1.40–2.98)	< 0.001	1.79 (1.23–2.63)	0.003	1.78 (1.20–2.65)	0.005

*Note* HR, hazard ratio; CI, confidence interval; PIM, potentially inappropriate medication

^a^ Hazard ratios are adjusted for sex, smoking history, tumor stage, ECOG PS, and CCI.

^b^ Hazard ratios are adjusted for age, sex, BMI, smoking history, tumor stage, ECOG PS, CCI, and the number of medications

### Association between the use of PIMs within six months following the initiation of systemic therapy and overall survival

**Fig. 2:**
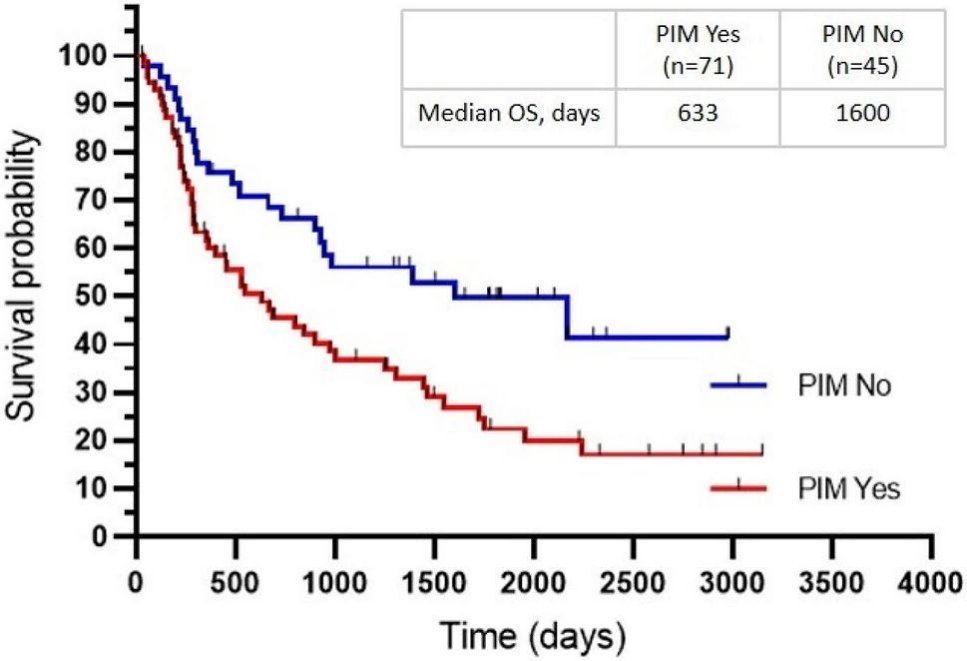
The Kaplan-Meier analysis of overall survival in NSCLC patients with or without PIM use within six months following the initiation of systemic therapy (*P* = 0.007). *Note* PIM, potentially inappropriate medication; OS, overall survival; NSCLC, non-small cell lung cancer

A total of 116 patients who had medication information were available for outcome analysis. PIM use within six months of the initiation of systemic therapy was associated with significantly lower OS in log-rank tests (median, 633 days vs. 1,600 days, *P* = 0.007; see Fig. 2).

In the multivariable analysis, PIM use within six months of the initiation of systemic therapy was associated with worse survival in adjusted analyses (model 1: adjusted HR, 1.86; 95% CI, 1.11–3.14; *P* = 0.020; model 2: adjusted HR, 1.79; 95% CI, 1.05–3.05; *P* = 0.032). Consistent with the observation of baseline PIM use, the use of two or more PIMs within six months of the initiation of systemic therapy was associated with poorer prognosis (multivariate analysis model 1: adjusted HR, 2.35; 95% CI, 1.31–4.21; *P* = 0.004; multivariate analysis model 2: adjusted HR, 2.43; 95% CI, 1.32–4.50; *P* = 0.005), whereas only the use of only one PIM was not (multivariate analysis model 1: adjusted HR, 1.48; 95% CI, 0.81–2.73; *P* = 0.206; multivariate analysis model 2: adjusted HR, 1.39; 95% CI, 0.75–2.58; *P* = 0.298; see Table 3).

**Table 3:** Univariate and multivariate Cox regression analyses of the association between PIM use within six months following the initiation of systemic therapy and overall survival

Variables	Univariate analysis		Multivariate model 1^a^		Multivariate model 2^b^	
	HR (95% CI)	*P*	HR (95% CI)	*P*	HR (95% CI)	*P*
PIM (yes vs. no)	1.97(1.19–3.26)	0.008	1.86(1.11–3.14)	0.020	1.79(1.05–3.05)	0.032
Number of PIMs **(1 vs. 0)**	1.56(0.86–2.82)	0.141	1.48(0.81–2.73)	0.206	1.39(0.75–2.58)	0.298
Number of PIMs (≥ 2 vs. 0)	2.50(1.42–4.39)	0.001	2.35(1.31–4.21)	0.004	2.43(1.32–4.50)	0.005

*Note* HR, hazard ratio; CI, confidence interval; PIM, potentially inappropriate medication

^a^ Hazard ratios are adjusted for sex, smoking history, tumor stage, CCI, and surgery

^b^ Hazard ratios are adjusted for age, sex, BMI, smoking history, tumor stage, ECOG PS, CCI, the number of medications, and surgery

## Discussion

Our study provided an important insight into the association between PIMs and OS in newly diagnosed NSCLC patients. In our cohort, PIM use was common among older patients with NSCLC. More importantly, PIM use negatively impacted patients’ OS and remained an independent predictor of poorer OS after adjusting for covariates.

It was reported that the prevalence of PIMs ranged from 28.4 to 45.0% in lung cancer patients [17], which was similar with that of baseline PIM use (39.9%) in our cohort, though our cohort showed a relatively higher prevalence of PIM use after the initiation of systemic therapy (61.2%). We investigated two distinct time points to analyze the changes in medication before and after the initiation of cancer therapy. Consistent with the results of Ortland and colleagues [22], PIM use was more common in older patients with cancer after initiation of cancer therapy, which in our study was primarily due to medications for the management of treatment-related symptoms or side effects, such as first-generation antihistamines and metoclopramide. In contrast, Karuturi et al. found a decreased prevalence of PIM use among older patients after the diagnosis of breast or colorectal cancers [23].

Several studies have examined the association between PIM use and patients’ clinical outcomes [16]. The pooled effect estimate suggested increased risks of hospitalization, prolongation of hospitalization, and treatment-related toxicity for PIM users, though this was not statistically significant [16]. Prior studies examining the association between the use of PIMs and mortality in older patients with malignancies found mixed results [11, 24–27]. The discrepancy in the results of these studies may be attributable to several reasons. First, patient populations included in these studies varied in the types of cancer they were diagnosed with. Common cancer types included breast cancer, colorectal cancer, non-Hodgkin lymphoma, and malignant hematology. However, none of the studies specifically investigated patients with lung cancer. Lung cancer has a median onset age of 70 years and the mortality in lung cancer, which accounts for ~ 20% of all cancer deaths, is high compared to other cancers [28, 29]. In addition, elderly NSCLC patients are naturally prone to PIMs due to the relatively high prevalence of comorbidity with aging or cancer-related symptoms [28, 29]. The senior average age at onset, high risk of PIM, and high mortality may all have contributed to the poor prognosis of NSCLC patients who were subject to PIM use. Second, PIM use was assessed at different times in these studies. The studies assessed PIM use at the time of diagnosis, surgery, hospital admission and discharge, and/or prior to hospital admission. Third, the variables considered in these studies were different and most studies did not control for comorbidities, which could have introduced confounding effects [30]. In addition, varying length of the follow-up period may also lead to inconsistent findings. Therefore, our study included only patients newly diagnosed with NSCLC and followed them up for nearly five years. In addition, we analyzed PIM use both at the time of diagnosis and within six months following the initiation of systemic treatment. To include as many confounding factors as possible and to ensure the reliability of the results of the multivariate analyses, we used two different models, one that considered only statistically significant variables and the other that considered both statistically and clinically significant variables. Our findings suggest that the use of PIMs that were identified based on the 2019 Beers criteria can negatively affect OS of older patients with NSCLC through following patients over a long period of time for survival, examining the use of PIMs at different time points, and comprehensively considering confounding factors in the multivariate analyses. We further explored the association between the number of PIMs used and OS and found that only the use of multiple PIMs conjunctively was an independent risk factor for poor cancer prognosis, suggesting that priority should be given to patients using multiple PIMs.

In line with our previous study that recruited older patients in general, PIM use was associated with the number of medications prescribed for older patients with NSCLC [21]. Previous studies investigating the Japanese population showed that polypharmacy was an independent prognostic factor in older patients with advanced NSCLC treated with ICI or epidermal growth factor receptor tyrosine kinase inhibitor (EGFR-TKI) [28, 29]. With increasing medication complexity in cancer patients, it is necessary to identify issues associated with polypharmacy for patients with cancer [31]. Considering the negative effect of PIM use on the prognosis of NSCLC patients, we suggest that deprescribing shall be incorporated into routine clinical practices and that medication reconciliation could be a viable strategy to reduce the incidence of PIM use among older adults with NSCLC.

Our study has the following strengths. First, the study results illustrated the impact of PIM use on the prognosis of elderly NSCLC patients, a population poorly investigated by previous studies. Second, we defined overall survival, the “golden-standard” endpoint in cancer clinical trials [32], as a prognostic indicator for patients with a long follow-up period. Third, we adjusted for a variety of confounding factors in the multivariable models to strengthen the reliability of our results.Our study also has several limitations. First, OS was the only prognostic indicator in this study and we did not evaluate the association between PIM use and other patient clinical outcomes such as hospitalization, quality of life, and treatment-related toxicity. Second, medication information was extracted from EMRs. As a result, medication information after the initiation of systemic therapy for some patients cannot be accessed as some patients were not readmitted to the hospital after diagnosis. Third, due to the limited sample size and the fact that cancer treatment regimens were not available for some patients, we only classified treatment methods based on whether or not surgery was performed, which may lead to an underestimation of the impact of treatment methods on prognosis. Fourth, this study was a single-center study. Therefore, our results may have potential biases, limiting the generalizability of our results. Our findings should be interpreted with caution due to the modest sample size. Fifth, we did not measure potential prescribing omissions, whose association with prognosis needs further research. Sixth, although we adjusted for many covariates, we cannot distinguish cases where PIM use was necessitated due to the patients’ refractory conditions.

## Conclusions

In conclusion, evidence generated from our cohort study indicates a high prevalence of PIM use in older NSCLC patients at Beijing Chao-Yang hospital in China and PIM use remains an independent risk factor for OS. Medication reconciliation and de-prescribing aimed at PIMs are needed in routine clinical practices to reduce the use of PIMs among older patients with NSCLC, with priority given to patients using multiple PIMs.

### Electronic supplementary material

Below is the link to the electronic supplementary material.

Supplementary Material 1

## Data Availability

The authors confirm that the data supporting the findings of this study are available within the article [and/or its supplementary materials].
