# Supplementary material for: Association of potentially inappropriate medications with prognosis among older patients with non-small cell lung cancer
Source: BMC Geriatr. 2024 Jun 25;24:550. doi: 10.1186/s12877-024-05138-3 (PMC11197362; doi:10.1186/s12877-024-05138-3)
Supplement: Supplementary file 1 — Supplementary Material 1 [file 12877_2024_5138_MOESM1_ESM.docx]

**Supplementary Table 1: The number of PIMs detected in NSCLC patients**

| **PIM use** | **Baseline (n=338)** | **Within six months following the initiation of systemic therapy (n=116)** | **P value^a^** |
| --- | --- | --- | --- |
| **At least one PIMs** | 135 (39.9%) | 71 (61.2%) | <0.001 |
| **1 PIM** | 90 (66.7%) | 34 (47.9%) | 0.576 |
| **≥2 PIMs** | 45 (33.3%) | 37 (52.1%) | <0.001 |

Abbreviations: PIM, potentially inappropriate medication; NSCLC, non-small cell lung cancer.

^a^ P value tests for differences of PIM use between at baseline and within six months following the initiation of systemic therapy.

**Supplementary Table 2: Top five PIMs based on the Beers criteria**

|  | **PIM items at baseline (N=209)** | |  | **PIM items within six months following the initiation of systemic therapy (N=124)** | |
| --- | --- | --- | --- | --- | --- |
| **NO.** | Drugs and items | N(%) |  | Drugs and items | N(%) |
| **1** | Diuretics | 57(27.3) |  | Diuretics | 37(29.8) |
| **2** | Benzodiazepines | 29(13.9) |  | First-generation antihistamines | 23(18.5) |
| **3** | Tramadol | 20(9.6) |  | Metoclopramide | 15(12.1) |
| **4** | Insulin, sliding scale | 19(9.1) |  | Benzodiazepines | 13(10.5) |
| **5** | Non–cyclooxygenase-selective NSAIDs | 10(4.8) |  | Non–cyclooxygenase-selective NSAIDs | 7(5.6) |
| **5** | Proton-pump inhibitors | 10(4.8) |  |  |  |

Abbreviations: PIM, potentially inappropriate medication; NSAIDs, non-steroidal anti-inflammatory drugs.

**Supplementary Table 3: Multivariate analysis of risk factors associated with PIM use**

| **Variables** | **Baseline (n=338)** | | **Within six months following the initiation of systemic therapy (n=116)** | |
| --- | --- | --- | --- | --- |
|  | OR (95% CI) | P value | OR (95% CI) | P value |
| **Age (≥74 vs. 65-74)** | 0.90(0.51-1.61) | 0.732 | 0.81(0.27-2.44) | 0.707 |
| **Sex (male vs. female)** | 0.75(0.46-1.24) | 0.267 | 1.24(0.50-3.06) | 0.638 |
| **BMI (≥25 vs. ＜25)** | 0.82(0.49-1.36) | 0.434 | 0.80(0.32-2.00) | 0.625 |
| **ECOG PS (3-4 vs. 0-2)** | 1.09(0.62-1.93) | 0.765 | 0.44 (0.15-1.33) | 0.147 |
| **CCI** | 0.10(0.92-1.08) | 0.963 | 1.05(0.91-1.21) | 0.507 |
| **Number of Medications** | 1.20(1.14-1.27) | < 0.001 | 1.24(1.12-1.37) | < 0.001 |

Abbreviations: OR, odds ratio; C, confidence interval; BMI, body mass index; ECOG PS, Eastern Cooperative Oncology Group Performance Status; CCI, Charlson Comorbidity Index; PIM, potentially inappropriate medication.
